# Obituary

**Published:** 1891-06

**Authors:** 


### Obituary.

Died, at his home in Philadelphia, Wednesday, May 27th, Dr. James W. White, President of the S. S. White Dental Manufacturing Co., and Editor of the *Dental Cosmos*, in the 65 year of his age.

Dr. White was born in Bucks county, Pa., in 1826. He had resided in Philadelphia since his boyhood, and graduated in medicine many years ago, but never practiced medicine as a vocation, but only for study and as an aid to his charitable work. He was the senior member of the firm of Hance Bros. & White, manufacturing chemists, and president of the largest enterprise of its class in the world, the S. S. White Dental Manufacturing Company, which employs more than 600 men and women in its several establishments, and with which he was identified for over forty years. His practical philanthropic work was large, but was done quietly and unassumingly.

Dr. White received his degree of M.D. from the University of Pennsylvania. The honorary degree of D.D.S. was conferred on him by the Pennsylvania College of Dental Surgery, and the degree of A.M. by the St. Lawrence University, of Canton, N. Y. For many years he was editor of *Dental Cosmos*.

In addition to his connection with the firms named he was a director of the German-American Title and Trust Company.

Dr. White married at Philadelphia, October 28, 1847, Mary Ann, daughter of James and Maria McClaranan. He leaves, besides his widow, three sons, Dr. J. William White, Professor of Clinical Surgery at the University of Pennsylvania; Samuel S. White, of the firm of Patterson & White, printers, and Louis P. White, wholesale jeweler.

Dr. White was a man of strong convictions and courageous in upholding his opinions. He was pre-eminently unselfish; personal sacrifice was to him a pleasure if it promoted the good of others. His relations with his employes were of the most kindly and pleasant nature. From the highest to the lowest in the great establishment of which he was the executive head, the feeling towards him among those employed was that of sincere friendship, and in his death they mourn a friend whose hand and counsel were always at their call.

The followeng details regarding his death have been made known by the family: In the winter he had a severe attack of a somewhat obscure trouble which was diagnosed as rheumatism of the throat. From this he recovered slowly, but its effects were gradually wearing away. Tuesday he complained somewhat of an uncomfortable feeling about the heart, but continued to attend to his duties, and was at the office throughout the day. Yesterday morning on arising he spoke of feeling "dyspeptic," and shortly afterwards, while adjusting his shoes, fell forward, dying in a few moments. Dr. W. D. Robinson was hastily summoned, but the end had come before he reached the house.

Dr. White was a member of the Universalist Church, and gave a large share of his time, money and talents to helping the poor and unfortunate of his city. He was a modest man, yet a man of the deepest convictions on moral subjects, and his earnest endeavors to be and do and have others do right have compelled him to take positions before the public that required great force of character to maintain. The profession is somewhat familiar with the controversy he, as president of the great dental supply house, was compelled to engage in with some of the eastern dentists, and no one, however he may differ from Dr. White, will say that he did not maintain his position with dignity and honor.

The profession is greatly indebted to Dr. White for, not only his interest in the welfare of the profession, but for his frequent contributions to its literature, and for what, as far as the dental profession perhaps is concerned, will be his monument, commemorating his life in its behalf, namely, *The Dental Cosmos*, a journal that represents the best there is in dental literature and is more widely read than any other professional journal. To his diligence and wisdom the *Cosmos* has lived up to its motto of "*observe, compare, reflect and record*," and the dental profession is the gainer. We all honor and revere his noble life, and most deeply sympathize with his bereaved kindred. H.

---

THE faculty of the Dental College of the University of Michigan adopted the following minute in regard to the death of Fred E. Spencer, of the class of '93. To Mr. E. R. Spencer, Dowagiac, Michigan.

*Dear Sir:*—The faculty of the college of Dental Surgery of the University of Michigan, wishes to express to you the sense of loss it has sustained by the death of your son, Mr. Fred E. Spencer of the class of 1893. His gentle bearing, his studiousness, his constant effort to excell made him not only a leader in a class containing so many kindred spirits, but a great helper to his instructors as well.

The faculty desires to express its heartfelt sympathy to you in this great affliction that we know you have sustained in his death.

Signed, J. TAFT, DEAN.
N. S. HOFF, Secretary.

---

DIED, in Chicago March 20th, 1891, of pneumonia, Dr. E. R. E. Carpenter in his fifty-ninth year.

Dr. Carpenter was born in Providence R. I., May 4th, 1832. He began the practice of dentistry in Chicago in 1860, where he devoted nearly all his professional life, he attained a very high reputation as an operator. He possessed a warm liberal hearted nature that was admired by all who knew him.

